# Effect of nutritional status on the ovarian follicular population, yield and quality of oocytes in the Ngaoundere Gudali zebu (*Bos indicus*)

**DOI:** 10.14202/vetworld.2015.502-507

**Published:** 2015-06-16

**Authors:** Justin Kouamo, Sorelle Gwladys Djatche Tidjou, Andre Pagnah Zoli, Youssouf Mouliom Mfopit

**Affiliations:** 1 Department of Surgery and Medical Pathology, School of Veterinary Medicine and Sciences, The University of Ngaoundere, Po Box 454, Ngaoundere, Cameroon; 2 Department of Physiology and Biotechnology of Reproduction, School of Veterinary Medicine and Sciences, The University of Ngaoundere, Po Box 454, Ngaoundere, Cameroon; 3 Department of Biochemistry, Regional Centre of the Institute of Agricultural Research for Development Wakwa, Po Box 65, Ngaoundere, Cameroon

**Keywords:** body condition score, energy, follicles, Gudali, oocytes, protein, phosphorus

## Abstract

**Aim:**

The aim of this study was to investigate the effect of nutritional status of the Gudali cows slaughtered at the Ngaoundere abattoir on follicular population, quality, and oocytes yield.

**Materials and Methods:**

Blood and ovaries were collected from 81 cows aged 6.35±0.24 years (3-12 years old), with a body condition score (BCS) of 2.93±0.09 (1-5). In each ovary, the follicle were counted and classified as small (<3 mm), medium (3-8 mm) and large (>8 mm) using an electronic caliper. Oocytes were collected by slicing technique and classified according to the homogeneity of the cytoplasm and layers of granulosa into four groups: I, II, III, and IV. The nutritional status of the animals was determined by quantification of serum glucose, total cholesterol, total protein, albumin, globulins, urea, and phosphorus level.

**Results:**

Of the total 162 ovaries harvested, 2916 follicles were counted on the ovarian surface with an average population of 36.00±2.17 follicles/cow. According to a size distribution, 16.67±1.54 (46.3%), 18.83±1.27 (52.3%), and 0.51±0.07 (1.4%), respectively for small (<3 mm), medium (3-8 mm), and large (>8 mm) were recorded. About 1,929 oocytes were obtained, with an average recovered of 23.81±1.53 oocytes/cow. Depending on the quality, 7.79±0.55 (32.7%), 6.04±0.41 (25.3%), 4.89±0.44 (20.6%), and 5.10±0.54 (21.4%) oocytes qualities I, II, III, and IV were obtained respectively; with an average cultivable oocyte recovered of 13.83±0.89 (58%). Cows with BCS > 3 and a high albumin and phosphorus level showed a highest number of follicles and oocytes able for *in vitro* maturation.

**Conclusion:**

These results indicated that nutrition remains an important factor for the *in vitro* production of the good embryo and the BCS is a useful tool for the selection of females’ oocytes donors.

## Introduction

The livestock sector is a major component of the agricultural economy of Cameroon and contributes to 950 billion FCFA to the national investment budget [1]. However, poor genetic makeup and reproductive failures limit the productivity of Cameroon livestock. Assisted reproductive technologies like artificial insemination (AI), superovulation, *in vitro* fertilization (IVF), embryo transfer (ET), *in vitro* embryo production (IVEP) [2] have been developed to overcome these problems, to increase the number of offspring from selected females, and to reduce the generation intervals in farm animals. Laboratory production of embryos (IVF technology) provides an excellent and cheap source of embryos for carrying basic research on developmental physiology, farm animal breeding, and for commercial application of the emerging biotechniques such as cloning and transgenesis [3].

In Cameroon, artificial insemination of local breeds with exotic semens (Friesian, Holstein, Brahman) has contributed for the improvement of cattle genetic potential [4]. This technique is used in certain dairy farms in the Northwest and Adamawa regions of Cameroon. However, embryo transfer and *in vitro* production of cattle embryo have not been tested to date in Cameroon. Indeed, in cattle, the IVEP is widely used in the world and has allowed the production of high genetic embryos from oocytes collected from live cows or cows slaughtered in order to promote the growth of this species [5]. Enormous progress in the production of bovine blastocysts *in vitro* has been made since the first calf was born from an *in vitro* fertilized embryo in 1981 [6]. The process of maturation, fertilization, and *in vitro* culture were significantly improved [3] to the point they are used in breeding programs and also in the development of laboratories in mass production of embryos *in vitro*. However, the egg donor intrinsic and extrinsic factors such as follicular size, morphology of oocyte cumulus complex, biochemical, hormonal, and nutritional aspects [7] have been reported as the cause of results fluctuations in the same laboratory.

It is in this context that this study was conducted with the main objective to study the effect of nutritional status on the follicular population, the yield and the quality of Gudali cows oocytes. This has been achieved by the determination of follicular population, quantitative and qualitative oocytes yield, the nutritional status of animals; and the impact of energy and protein parameters, and phosphorus on the follicular population and oocytes yield.

## Materials and Methods

### Ethical approval

The experimental protocol followed the Ethical guidelines on the proper care and use of animals and had been approved by the ministry of livestock, fisheries, and animal industry.

### Study area and animal selection

The work was carried out in Ngaoundere, Adamawa region, Cameroon (Latitude19°7’39N and Longitude13° 35’4 E), characterized by a Guinea Savana climate. The samples were collected at the Ngaoundere municipal slaughterhouse from December to April and analyzed in the Veterinary Research Laboratory of IRAD Wakwa and food technology laboratory of ENSAI (National school of industrial agro sciences of the University of Ngaoundere).

The study was conducted on 81 female Gudali zebu aged from 3 to 12 years old (6.35±0.24), with an average body condition score (BCS) of 2.93±0.09 (27.1%, 51.9%, and 21% animals, respectively, for thin [1-2 BCS], normal [3 BCS], and fat [4-5 BCS]).

### Blood collection

Blood from jugular vein was collected aseptically from each Gudali (Venoject^®^). After collecting blood, the serum was separated out by centrifugation (2700 g, 15 min, 4°C), and aliquots were stored at −20°C until assayed.

### Harvest of ovaries and assessment of follicular population

Ovaries (162) were obtained within 2 h [8] of slaughter from a municipal Ngaoundere abattoir and were transported to the laboratory in a thermos flask containing sterile warm (34-36°C) physiological normal saline solution supplemented with antibiotics (0.5 mg/ml penicillin-streptomycin sulfate). All ovaries were cleared off the attached tissue and mesovarium (trimming). The trimmed ovaries were subject to washings (5-6 times) with warm saline fortified with antibiotics and transferred into the laminar flow. All subsequent experimental procedures were conducted in laminar flow. The apparent follicles on each ovary were measured using an electronic caliper then counted and classified according to their diameter into small (<3 mm), medium (3-8 mm), and large (>8 mm) follicles [9].

### Oocytes recovery

Oocytes were harvested by slicing technique: ovaries were placed in a graded plastic Petri dish containing oocyte collection medium (Dulbecco’s phosphate-buffered saline) and were chopped into small pieces with a surgical blade [10]. The collected oocytes (1929) were finally graded as excellent (I), good (II), fair (III), and poor (IV) quality under the stereo microscope (×10) depending on their cumulus investment and cytoplasmic distribution: Grade I: Oocytes with more than 4 layers of bunch of compact cumulus cells mass with evenly granulated cytoplasm; Grade II: oocyte with at least 2-4 layers of compact cumulus cell mass with evenly granulated cytoplasm; Grade III: Oocyte with at least 1 layer of compact cumulus cell mass with evenly granulated cytoplasm; Grade IV: Oocyte with no cumulus cells or incomplete layer of cumulus cell or expanded cells, and having dark or unevenly granulated cytoplasm [11].

### Biochemical analysis

Serum sample was evaluated for the concentration of glucose, total cholesterol, urea, total protein, albumin, and phosphorus. Total globulins were calculated by subtracting albumin from total protein [12]. All biochemical parameters were determined by a spectrophotometric method using commercial kits supplied by Cromatest^®^.

### Statistical analysis

Data obtained were recorded and statistically analyzed using Statistical Package for Social Science software version 20. Analysis of Variance and Turkey HSD tests were used to compare different groups. The level of significance was recorded at the 5% level of confidence.

## Results

### Follicular population

Of the total 162 ovaries collected from 81 cows, 2916 follicles of all sizes were counted on the surface with an average follicular population of 36.00±2.17 follicles/cow. Depending on the size, the average population of small (<3 mm), medium (3-8 mm), and large (>8 mm) follicles was 16.67±1.54 (46.3%), 18.83±127 (52.3%), and 0.51±0.07 (1.4%), respectively.

### Recovery rate

**Figure-1:**
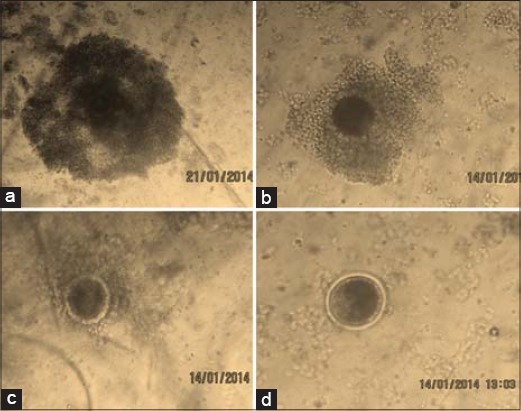
Oocytes (a) Grade I, (b) Grade II, Grade III, (d) Grade IV.

From 81 pairs of ovaries, 1929 oocytes were obtained with an average yield of 23.81±1.53 oocytes/cow. Depending on the quality, 7.79±0.55 (32.7%), 6.04±0.41 (25.3%), 4.89±0.44 (20.6%), and 5.10±0.54 (21.4%) oocytes were of quality I, II, III, and IV, respectively (Figure-1), with an average selected oocytes for IVEP of 13.83±0.89 (58%).

### Global nutritional status of animals

Biochemical variables assayed in 81 cows’ serum collected for this study was grouped in energy parameters (glucose and total cholesterol), protein parameters (total protein, albumin, globulin, and urea), and phosphorus. Table-1 shows the nutritional status of zebu cows Gudali studied. The majority of animals (over 50%) have concentrations within the normal range.

**Table-1:** Distribution of the cows according to their nutritional state.

Nutritional parameters	Mean±SEM (min-max)	Blood concentration (%)			Reference values*

		Low	Normal	High	
Glucose	5.70±0.26 (2.72-14.47)	0 (0)	41 (50.6)	40 (49.4)	2.6-4.9 mmol/l
Total cholesterol	6.20±0.29 (2.24-20.34)	0 (0)	42 (51.9)	39 (48.1)	2.3-6.0 mmol/l
Total protein	65.48±1.19 (39.95-92.64)	26 (32.1)	46 (56.8)	9 (11.1)	59.5-80 g/l
Albumin	35.18±0.81 (13.05-49.80)	11 (13.5)	51 (64)	19 (23.5)	27.7-40.4 g/l
Globulins	30.30±1.37 (4.10-57.20)	30 (37)	44 (54.3)	7 (8.6)	26.2-45.5 g/l
Urea	4.79±0.23 (1.94-12.20)	32 (39.5)	32 (39.5)	17 (21)	3.8-6.5 mmol/l
Phosphorus	2.01±0.07 (0.88-4.77)	3 (3.7)	72 (88.9)	6 (7.4)	1.05-2.83 mmol/l

* Kouamo et al., 2011; SEM=Standard error of means, Min=Minimum value, Max=Maximum value

### Effect of nutritional parameter on follicular population and oocytes recovery rate

Table-2 presents the effect of the BCS, the serum concentration of glucose, total cholesterol, total protein, albumin, globulins, and phosphorus on the follicular population, oocyte yield and quality in the Gudali zebu. It showed that cows with BCS of 4-5, high level of albumin and phosphorus had the best follicular population and oocyte yield.

**Table-2:** Effect of nutritional parameters on follicular population and oocyte recovery rate.

Blood parameters	Follicles categories				Oocytes					
	
	Average number of follicles/animal	Small (<3 mm)	Medium (3-8 mm)	Large (>8 mm)	Average oocytes recovered/animal	Grade I	Grade II	Grade III	Grade IV	Selected oocytes for IVEP (I+II)
BCS										
1-2	27.41±3.80^a^	13.27±1.96^a^	13.64±2.30^a^	0.50±0.14^a^	15.86±2.64^a^	5.91±1.15^a^	4.45±0.77^a^	2.27±0.47^a^	3.23±1.03^a^	10.36±1.85^a^
3	38.60±2.68^b^	15.88±2.03^a^	22.17±1.67^b^	0.55±0.09^a^	26.02±1.65^b^	8.33±0.59^a^	6.76±0.51^b^	5.38±0.52^b^	5.55±0.64^a^	15.10±0.9^b^
4-5	40.71±5.78^b^	23±4.53^b^	17.29±2.80^ab^	0.41±0.17^a^	28.65±4.398^b^	8.88±1.50^a^	6.29±0.96^b^	7.06±1.39^b^	6.41±1.41^a^	15.18±2.34^b^
p value	0.023	0.08	0.013	0.77	0.004	0.103	0.051	0.000	0.084	0.05
Glucose (mmol/l)										
N	37.22±3.07^a^	18.34±2.43^a^	18.41±1.71^a^	0.46±0.28^a^	25.02±2.24^a^	7.76±0.76^a^	5.60±0.51^a^	4.90±0.60^a^	5.90±0.84^a^	14.22±1.30^a^
H	34.75±3.09^a^	14.95±1.88^a^	19.25±1.91^a^	0.55±0.32^a^	22.58±2.09^a^	7.83±0.80^a^	6.46±0.63^a^	4.88±0.70^a^	4.28±0.64^a^	13.43±1.21^a^
p value	0.57	0.27	0.74	0.55	0.42	0.95	0.29	0.97	0.13	0.65
Total cholesterol (mmol/l)										
N	32.26±2.42^a^	14.98±1.60^a^	16.76±1.51^a^	0.52±0.10^a^	22.86±2.01^a^	7.60±0.65^a^	5.95±0.58^a^	4.45±0.55^a^	4.85±0.72^a^	13.50±1.15^a^
H	40.03±3.59^a^	18.49±2.70^a^	21.05±2.05^a^	0.49±0.10^a^	24.85±2.47^a^	8.00±0.91^a^	6.13±0.60^a^	5.36±0.74^a^	5.36±0.74^a^	14.13±1.37^a^
p value	0.07	0.26	0.09	0.80	0.52	0.71	0.83	0.32	0.64	0.74
Total proteins (g/l)										
L	35.58±3.40^a^	15.69±2.39^a^	19.38±1.93^a^	0.50±0.11^a^	24.23±2.87^a^	8.19±1.07^a^	6.58±0.98^a^	4.69±0.72^a^	4.77±0.97^a^	14.77±1.75^a^
N	36.00±3.06^a^	16.04±1.85^a^	18.20±1.83^a^	0.57±0.11^a^	23.11±2.03^a^	7.67±0.74^a^	5.72±0.54^a^	4.63±0.61^a^	5.09±0.66^a^	13.39±1.19^a^
H	43.33±6.46^a^	22.67±7.73^a^	20.44±3.87^a^	0.22±0.15^a^	26.22±4.23^a^	7.22±0.92^a^	6.11±0.82^a^	6.78±1.70^a^	6.11±2.21^a^	13.33±1.32^a^
p value	0.48	0.39	0.83	0.35	0.81	0.86	0.64	0.34	0.77	0.76
Albumin (g/l)										
L	21.91±3.86^a^	7.55±1.72^a^	13.73±3.07^a^	0.64±0.20^a^	14.45±3.14^a^	5.45±1.21^a^	4.27±1.07^a^	2.09±0.56^a^	2.64±0.79^a^	9.73±2.17^a^
N	37.98±2.86^b^	18.02±2.09^b^	19.53±1.77^a^	0.43±0.09^a^	25.57±1.94^b^	8.33±0.71^a^	6.14±0.51^a^	5.25±0.63^b^	5.84±0.73^a^	14.47±1.14^a^
H	38.84±4.00^b^	18.32±2.93^b^	19.89±1.84^a^	0.63±0.14^a^	24.53±3.16^b^	7.68±1.09^a^	6.79±0.78^a^	5.53±0.78^b^	4.53±1.02^a^	14.47±1.75^a^
p value	0.03	0.06	0.28	0.41	0.05	0.21	0.18	0.05	0.11	0.18
Globulins (g/l)										
L	35.50±3.13^a^	16.33±2.15^a^	18.67±1.61^a^	0.50±0.12^a^	24.37±2.67^a^	8.00±0.94^a^	6.47±0.66^a^	5.07±0.65^a^	4.83±0.89^a^	14.47±1.50^a^
N	36.64±3.05^a^	16.61±1.92^a^	19.52±2.02^a^	0.50±0.11^a^	24.07±2.03^a^	8.07±0.76^a^	5.82±0.57^a^	4.84±0.65^a^	5.34±0.69^a^	13.89±1.23^a^
H	34.14±10.14^a^	18.43±10.22^a^	15.14±3.28^a^	0.54±0.20^a^	19.86±5.66^a^	5.14±0.96^a^	5.57±1.23^a^	4.43±2.01^a^	4.71±2.49^a^	10.71±2.04^a^
p value	0.94	0.94	0.65	0.96	0.73	0.33	0.71	0.93	0.88	0.53
Urea (mmol/l)										
L	36.59±3.37^a^	18.94±2.59^a^	18.13±1.70^a^	0.53±0.11^a^	23.69±2.43^a^	7.72±0.94^a^	5.94±0.54^a^	5.00±0.85^a^	5.03±0.79^a^	13.66±1.37^a^
N	36.25±3.90^a^	15.28±2.29^a^	20.41±2.15^a^	0.50±0.13^a^	25.22±2.47^a^	8.47±0.81^a^	6.75±0.74^a^	5.16±0.69^a^	4.84±0.83^a^	15.22±1.40^a^
H	34.41±5.33^a^	16.88±3.55^a^	17.18±3.27^a^	0.35±0.15^a^	21.41±3.40^a^	6.65±1.21^a^	4.88±0.84^a^	4.18±0.76^a^	5.71±1.42^a^	11.53±2.02^a^
p value	0.93	0.75	0.59	0.55	0.65	0.47	0.23	0.72	0.83	0.30
Phosphorus (mmol/l)										
L	27±8.74^a^	16.67±5.21^a^	9.67±4.18^a^	0.67±0.33^a^	20.33±11.67^a^	7.33±4.06^a^	4.00±2.00^a^	2.67±2.19^a^	6.63±3.76^a^	11.33±5.21^a^
N	34.68±2.23^a^	15.39±1.59^a^	18.78±1.38^ab^	0.51±0.08^a^	22.49±1.52^a^	7.31±0.56^a^	5.64±0.40^a^	4.74±0.48^a^	4.81±0.56^a^	12.94±0.88^a^
H	56.33±7.50^b^	32.00±5.29^b^	24.00±2.89^b^	0.33±0.21^a^	41.50±3.86^b^	18.83±0.95^b^	11.83±0.79^b^	7.83±1.58^a^	8.00±2.10^a^	25.67±0.88^b^
p value	0.022	0.017	0.21	0.74	0.004	0.006	0.000	0.13	0.27	0.000

In each column different letters (a,b) indicated significant between group (p<0.05). L=Low; N=Normal; H=High, IVEP=*In vitro* embryo production

## Discussion

### Follicle categories

Studies have shown that follicle size increases with oocyte quality [13]. The average number of follicles counted per cow was higher than 16.75±1.05 and 32 reported by Acar *et al*. [9] and Takaji *et al*. [14], respectively. The average number of small, medium, and large follicles was different from the 12.10±1.06; 4.30±0.46, and 0.35±0.10, respectively reported by Acar *et al*. [9] in Turkey. The number of medium-sized follicles per cow was higher than that (5.20) obtained by Kumar *et al*. [15], but lower than 22.98±8.41 reported by Fihri [16] suggesting that there may be a breed [15,17] and/or a herd management effects. Fihri [16] reported that the Blonde Moroccan local breed in Oulmès-Zaer offer fewer follicles (18.96±1.3) than Holstein (25.19±1.63) and their crossbreed (24.71±1.69). In addition, the cow parity (number of calving) could be the cause of variation in ovarian dynamics [17].

### Oocytes recovery

The recovery of a large number of oocytes with high developmental competence remains an ultimate goal for the mass production of embryos in cattle. The obvious advantages needed for a good production of embryo are the speed of oocytes recovery, technique used, and quality and quantity of oocytes [13]. The average number of selected oocytes for IVEP by slicing are much higher than 2.59±1.53 reported by Fihri [16], 8.00±1.53 by Acar *et al*. [9], 9.6±0.4 by Wang *et al*. [18], but lower than the 55, 66 and 93 obtained by Carolan *et al*. [19]; Carolan *et al*. [20] and Mogas *et al.*, [21] respectively. The lower number of oocytes obtained by slicing in this study may be due to the slicing techniques that used in this study (each ovary was chopped into small pieces with a surgical blade), while the other researchers used blades to incise the follicles on the ovarian surface. A number of oocytes, therefore, were retained in ovary without recovery or were disintegrated due to injuring during chopping [18]. In addition to this technical factor, breed and nutrition could have affected the oocyte yield [16].

### Relation between nutritional status of Gudali, follicular population, and oocytes recovery

The average BCS obtained is comparable to those reported by Fihri [16] and Bah *et al*. [22] in Morocco and Ngaoundere slaughterhouses, respectively. The significant nutritional effect observed was also found in the Blonde Moroccan local breed of Oulmès-Zaer [16]. In the montbeliard cows, Delacharlerie [23] showed that BCS influenced significantly the oocytes production while cows with BCS ≥3 (normal cows or fat) were the best donors having transferable embryos. Pushpakumara *et al*. [24] showed that low BCS (≤2) negatively influenced both the number and quality of oocytes. BSC had a positive influence on the follicular population in accordance with other findings [25]. Thin cows (BCS ≤2) have fewer developing follicles during the luteal phase of the estrous cycle and tend to produce fewer eggs during the follicular phase compared to cows with BCS ≥3 [26].

Energy balance and the ability to mobilize fat reserves have an important role in reproductive function and especially on ovarian dynamics [27]. According to Cassidy *et al*. [28], cows with energy deficiency had poor follicular growth and those fed on diets rich in energy had increased rate of follicular growth [29]. A positive correlation (r=0.41) was reported by Rodriguez *et al*. [30] between the level of cholesterol and oocyte yield. Garnsworthy *et al*. [27] showed that a diet rich in starch stimulated follicles development and increased ovulation rate before day 50 post-partum. In this study, there was no significant correlation between the energy parameters (glucose, cholesterol) and the follicles, quantity, and oocyte quality. The sampling was done within the same period with the availability of good quality forage rich in energy values consequently all the animals were at the same energy level.

Protein provides amino acids that are necessary for the maintenance of vital functions: growth, reproduction, and lactation while serum urea is an indicator of the balance between nitrogen and energy intake. Protein intake affects cows’ reproduction through direct effects on the functioning of the corpus luteum (decrease progesterone level) and particularly the uterine environment where byproducts of nitrogen metabolism including rumen ammonia can compromise the survival of sperm, ovum or embryo [31]. There was no significant effect (p>0.05) of total protein, globulin or uremia on the follicle and oocyte studied. However, serum albumin had a significant effect (p<0.05) on the number of follicles and oocytes recovered. Follicular population and number of oocytes decrease when the albumin is lower than 27.7 g/l [16].

There was a significant effect of phosphorus on the number of follicles (p<0.05) and high significant effect on oocytes yield suitable for *in vitro* maturation (p<0.001). This suggests the importance of phosphorus to the reproductive function especially at ovarian level. Phosphorus deficiency induces direct disturbance of the reproductive function [32].

## Conclusion

This study indicated that for the practice of IVEP in Cameroon, the BCS is a useful tool for the selection of females’ oocytes donors. In addition, the serum phosphorus level could be an important supplementary analysis to distinguish cows with good quality oocytes. The selected cows should not suffer for undernutrition because it could adversely influence oocyte yield.

## Authors’ Contributions

JK designed the experiments and approved the experimental protocol. SGDT collected data from the area study. JK, SGDT, APZ, and YMM reviewed the manuscript. All authors read and approved the final manuscript.
